# Aberrant methylation of DACT1 and DACT2 are associated with tumor progression and poor prognosis in esophageal squamous cell carcinoma

**DOI:** 10.1186/s12929-016-0308-6

**Published:** 2017-01-11

**Authors:** Yan-li Guo, Bao-En Shan, Wei Guo, Zhi-Ming Dong, Zhen Zhou, Su-Peng Shen, Xin Guo, Jia Liang, Gang Kuang

**Affiliations:** Laboratory of Pathology, Hebei Cancer Institute, The Fourth Hospital of Hebei Medical University, Jiankanglu 12, Shijiazhuang, 050011 Hebei China

**Keywords:** Esophageal squamous cell carcinoma, *DACT* gene, Methylation, Prognosis

## Abstract

**Background:**

The *DACT* (Dishevelled-associated antagonist of β-catenin) family of scaffold proteins may play important roles in tumorigenesis. However, the epigenetic changes of *DACT1,* 2, 3 and their effect on esophageal squamous cell carcinoma (ESCC) have not been investigated so far. The aim of this study was to investigate the promoter methylation and expression of *DACT* family, in order to elucidate more information on the role of *DACT* with regard to the progression and prognosis of ESCC.

**Methods:**

MSP and BGS methods were respectively applied to examine the methylation status of *DACT*; RT-PCR, Western blot and immunohistochemistry methods were respectively used to determine the mRNA and protein expression of *DACT*; MTT, Colony-formation and Wound-healing assay were performed to assess the effect of *DACT1* and *DACT2* on proliferation and migration of esophageal cancer cells.

**Results:**

Frequent reduced expression of *DACT1, DACT2* and *DACT3* were found in esophageal cancer cell lines and the expression levels of *DACT1* and *DACT2* were reversed by 5-Aza-Dc. Decreased mRNA and protein expression of *DACT1* and *DACT2* were observed in ESCC tumor tissues and were associated with the methylation status of transcription start site (TSS) region. The hypermethylation of CpG islands (CGI) shore region in DACT1 was observed both in tumor and corresponding adjacent tissues but wasn’t related to the transcriptional inhibition of *DACT1*. The methylation status of TSS region in DACT1 and DACT2 and the protein expression of *DACT2* were independently associated with ESCC patients’ prognosis.

**Conclusions:**

The TSS region hypermethylation may be one of the main mechanisms for reduced expression of *DACT1* and *DACT2* in ESCC. The simultaneous methylation of *DACT1* and *DACT2* may play important roles in progression of ESCC and may serve as prognostic methylation biomarkers for ESCC patients.

**Electronic supplementary material:**

The online version of this article (doi:10.1186/s12929-016-0308-6) contains supplementary material, which is available to authorized users.

## Background

Esophageal cancer is the eighth most common malignancy in the world and the overall prognosis for esophageal cancer patients is very poor [1–3]. According to the WHO report in 2012, the world’s new cases of esophageal cancer were 455 thousands, and 223 thousands of them occurred in China, accounting for 49% [4]. In china, esophageal squamous cell carcinoma (ESCC) was the dominant histological type of esophageal malignances. Based on two national mortality surveys conducted in 1970s and 1990s, ESCC has a striking geographic distribution in China, with higher prevalence in Taihang mountain areas of North China [5]. There was also a strong tendency toward familial aggregation of ESCC in these high-risk areas [6], suggesting that multiple genetic and epigenetic events may contribute to the occurrence and progression of ESCC. However, the precise molecular mechanisms of the development and progression of ESCC still remained unknown. Therefore, additional elucidation of the molecular mechanisms involved in ESCC and the discovery of early detect biomarkers are urgently needed for more effective chemoprevention or treatment.

The *DACT* (Dishevelled-associated antagonist of β-catenin) family of scaffold proteins, including *DACT1, DACT2* and *DACT3*, were isolated by a screen for proteins interacting with Dishevelled protein central to Wnt signaling [7–9]. *DACT* encode a series of vertebrate intracellular proteins those can regulate intercellular signaling pathways [10–12]. According to previous reports, DACT proteins act as tumor suppressors in different tumor types [13–15]. In addition, decreased expression and promoter hypermethylation of *DACT1* and *DACT2* have been found in some primary tumors and tumor cell lines, including hepatocellular carcinoma [15], breast cancer [16], lung cancer [17], colorectal cancer [18] and some other cancers. However, Schussel et al. [19] showed quite a different result which the promoter methylation of *DACT1* and *DACT2* may not be a common event in oral squamous cell carcinoma. Although the promoter of *DACT3* also has a large CpG Island, but the study in colorectal cancer indicates that the histone modification, rather than the aberrant, may be the main regulated mechanism for inactivation of this gene [18]. To our best knowledge, the regulation mechanism of *DACT1, DACT2* and *DACT3* and their function in ESCC remains unknown. In the present study, we attempted to detect the role and methylation status of *DACT1, DACT2* and *DACT3* in ESCC, and to elucidate the effect of their methylation status on progression / prognosis of ESCC patients.

## Methods

### Cell culture and treatment

The human esophageal cancer cell lines TE1, TE13, T.Tn and Eca109 were cultured in RPMI-1640 medium (Invitrogen, Carlsbad, CA, USA) supplemented with 10% heat-inactivated fetal bovine serum (FBS, Invitrogen, Carlsbad, CA, USA), 100 U/mL penicillin, and 100 μg/mL streptomycin. Treatment of these four cells with DNA methyltransferase inhibitor 5-aza-2′-deoxycytidine (5-Aza-Dc, 5 μmol/L, Sigma, St Louis, MO, USA) and histone deacetylase (HDAC) inhibitor trichostatin A (TSA,0.3 μmol/L, Cayman Chemical Co, Ann Arbor, MI, USA) was performed as previously reports [20, 21]. Control cells received no drug treatment. DNA, RNA and protein were isolated from these cells.

### Patients and specimens

One hundred fifty-nine paired primary tumor and corresponding adjacent non-cancerous tissues of ESCC were collected from the Fourth Affiliated Hospital, Hebei Medical University between the years of 2005 and 2009. All procedures performed in this study were in accordance with the ethical standards of the institutional research committee and with the 1964 Helsinki declaration and its later amendments or comparable ethical standards. The study was approved by the ethics committee of Hebei Medical University Fourth Affiliated Hospital, and the informed consent was obtained from all of the patients. All study subjects were ethnically homogeneous Han nationality and were from the same areas, which were the high-risk areas of upper gastrointestinal cancers (UGIC) in Hebei province. The incidence of UGIC in this area was over one hundred per one hundred thousands [22, 23]. Complete clinicopathologic characteristics and UGIC family history of these cases were available before operation and during follow-up. Individuals with at least one first-degree relative or at least two second-degree relatives having esophageal/cardia/gastric cancer were defined as having family history of UGIC. Distribution of clinicopathological data in the study cohort was shown in Table 1. For this study, all of the subjects were re-examined and confirmed by professional pathologists for histopathological diagnosis. All the esophageal cancer were squamous cell carcinoma and the adjacent non-cancerous tissues were normal tissues or hyperplasia tissues, which confirmed by microscope examination. The survival data were ascertained through the Tumor Registry and Hospital chart review. Fourteen patients were lost to follow up.

**Table 1:** Protein expression of DACT1, DACT2 and DACT3 in ESCC Cases

Group	N	DACT1 Protein		DACT2 Protein	
		positive n (%)	*p*	positive n (%)	*p*
Age					
<60	74	44(59.5%)	0.935	37(50.0%)	0.337
≥60	85	50(58.8%)		49(57.6%)	
Gender					
Male	93	56(60.2%)	0.739	49(52.7%)	0.674
Female	66	38(57.6%)		37(56.1%)	
Histological grade					
Well/moderate	98	58(59.2%)	0.983	54(55.1%)	0.483
poor	61	36(59.0%)		32(52.5%)	
clinical stage					
I + II	86	59(68.6%)	0.008	53(61.6%)	0.038
III + IV	73	35(47.9%)		33(45.2%)	
Lymph node					
+	121	66(54.5%)	0.036	57(47.1%)	0.105
-	38	28(73.7%)		29(76.3%)	
Family history of UGIC					
+	61	31(50.8%)	0.093	26(42.6%)	0.022
-	98	63(64.3%)		60(61.2%)	

### Stable gene transfections

Transfection was done using FuGENE HD transfection reagent (Roche, Swiss) as recommended by the manufacturer’s instructions. Full-length *DACT1* and *DACT2* cDNA were cloned into pcDNA3.1 and pCMV6 vector. Then, exponentially grown TE1 and TE13 cells were transfected with *DACT1* and *DACT2* expression plasmid (pcDNA3.1-DACT1 and pCMV6-DACT2) or the relevant empty vector (pcDNA3.1/TE1 and pCMV6/TE1) as control. The resistant cell clones were selected with G418 expanded for further characterization.

### Cell proliferation assay

Cell proliferation was measured with MTT assay. The TE1 or TE13 cells, the stable transfected cells (pcDNA3.1-DACT1/TE1,TE13 and pCMV6-DACT2/TE1,TE13) and the cells treated with 5-Aza-Dc for 72 h were seeded in 96-well plates (3 × 10^3^) in 200 μl of RPMI 1640 with 10% FBS under standard culture conditions (six wells/group). MTT (Sigma-Aldrich, Saint Louis, MO, USA) in PBS (5 mg/ml) was added (20 μl /well) and plates were incubated at 37 °C for 4 h, after which culture media was removed, DMSO was added and the absorbance was measured at 570 nm. This experiment was repeated three times.

### Colony formation assay

For colony assay, The TE1 or TE13 cells, the stable transfected cells (pcDNA3.1-DACT1/TE1,TE13 and pCMV6-DACT2/TE1,TE13) and the cells treated with 5-Aza-Dc for 72 h were seeded at density 500 cells/35 mm dish (three dishes/group), and incubated in normal growth medium two weeks later, cell colonies were stained with hematoxylin and counted.

### Wound-healing assay

The TE1 or TE13 cells, the stable transfected cells (pcDNA3.1-DACT1/TE1,TE13 and pCMV6-DACT2/TE1,TE13) and the cells treated with 5-Aza-Dc for 72 h were seeded in 24-well plates at the concentration of 2 × 10^5^ per well and allowed to form a confluent monolayer for 24 h, and then scratched with a pipette tip and washed to remove floating cells. Cells were photographed at the same field every 2 h.

### *DACT1, DACT2, DACT3* mRNA expression via semi-quantitative reverse transcription-polymerase chain reaction (RT-PCR) and quantitative real-time RT-PCR assays

Total RNA was isolated from cell lines and tissues by Trizol reagent (Invitrogen, Carlsbad, CA) according to the manufacturer’s instructions. Agarose gel electrophoresis and spectrophotometric analysis (A260: 280 nm ratio) were used to evaluate RNA quality and quantity. RT-for-PCR kit (Clontech, Palo Alto, CA) with random priming as recommended in the protocol provided. All primers and reaction conditions are listed in Additional file 1: Table S1. The glyceraldehyde-3-phosphate dehydrogenase (GAPDH) gene was used as an internal control. For RT-PCR, the PCR products were separated in 2% agarose gel in electrophoresis and visualized with ethidium bromide staining, and quantified using an image analysis system (Gel work-2ID). Real time PCR reactions were performed by the Stepone Plus Thermal Cycler (Applied Biosystems, Foster City, CA, USA) and SYBR green PCR Master Mix (Life Technology, Foster City, CA, USA). The expression levels of target genes were normalized with GAPDH using the 2^-△△CT^ method [24]. The reaction was repeated in triplicate with each of the samples for quality control.

### Western blot analysis

Western blot was performed according to previous report [25]. Whole cell lysates from cell lines were prepared by lysing the cells in ice-cold RIPA buffer. The protein concentration was determined using the BCA Protein Assay Kit (Pierce, Rockford, IL, USA), and then, equal amount of proteins were separated by SDS-PAGE and electroblotted onto PVDF membranes (Millipore, Billerica, MA, USA). The membranes were blocked in 5% nonfat milk and incubated with Rabbit anti-human polyclonal antibody for DACT1 (1 μg/ml, ab72078, Abcam, UK) and DACT2(2 μg/ml, ab79042, Abcam, UK), and mouse anti-human monoclonal antibody for β-actin (1:5000 dilution, ab6276, Abcam, UK) and visualization with enhanced chemiluminescence (ECL) detection reagents (Beyotime, Haimen, Jiangsu, China).

### DACT1, DACT2 protein expression via immunohistochemical staining (IHC)

The protein expression of *DACT1, DACT2* and *DACT3* in tumor and corresponding adjacent tissues of ESCC was detected using the immunostaining method of avidin-biotin complex immunoperoxidase [26]. After blocking of endogenous peroxidase and non-specific reactions, the slides were incubated with rabbit anti-human polyclonal antibody for *DACT1* (1:100 dilution, abcam, UK), *DACT2* (1:100 dilution, abcam, UK) and *DACT3* (1:50 dilution, AmyJet Scientific), and then incubated with biotinylated secondary antibody and ABC reagent. 3, 3′-Diaminobenzidine (Sigma, St Louis, MO) was used as the chromogen, and counterstaining was done using hematoxylin. In negative controls, the primary antibody was replaced with nonimmune serum for the negative control. Slides with positive staining of *DACT1, DACT2* and *DACT3* were used as positive control. Protein expression of *DACT1, DACT2* and *DACT3* were evaluated according to a scoring method reported previously [26]. Scoring accounted for both intensity and the percentage of positive cells. All slides were examined and scored by three independent observers, who were blinded to the clinical data.

### Methylation analysis of *DACT* via methylation-specific polymerase chain reaction (MSP) and bisulfite genomic sequencing (BGS) methods

**Fig. 1:**
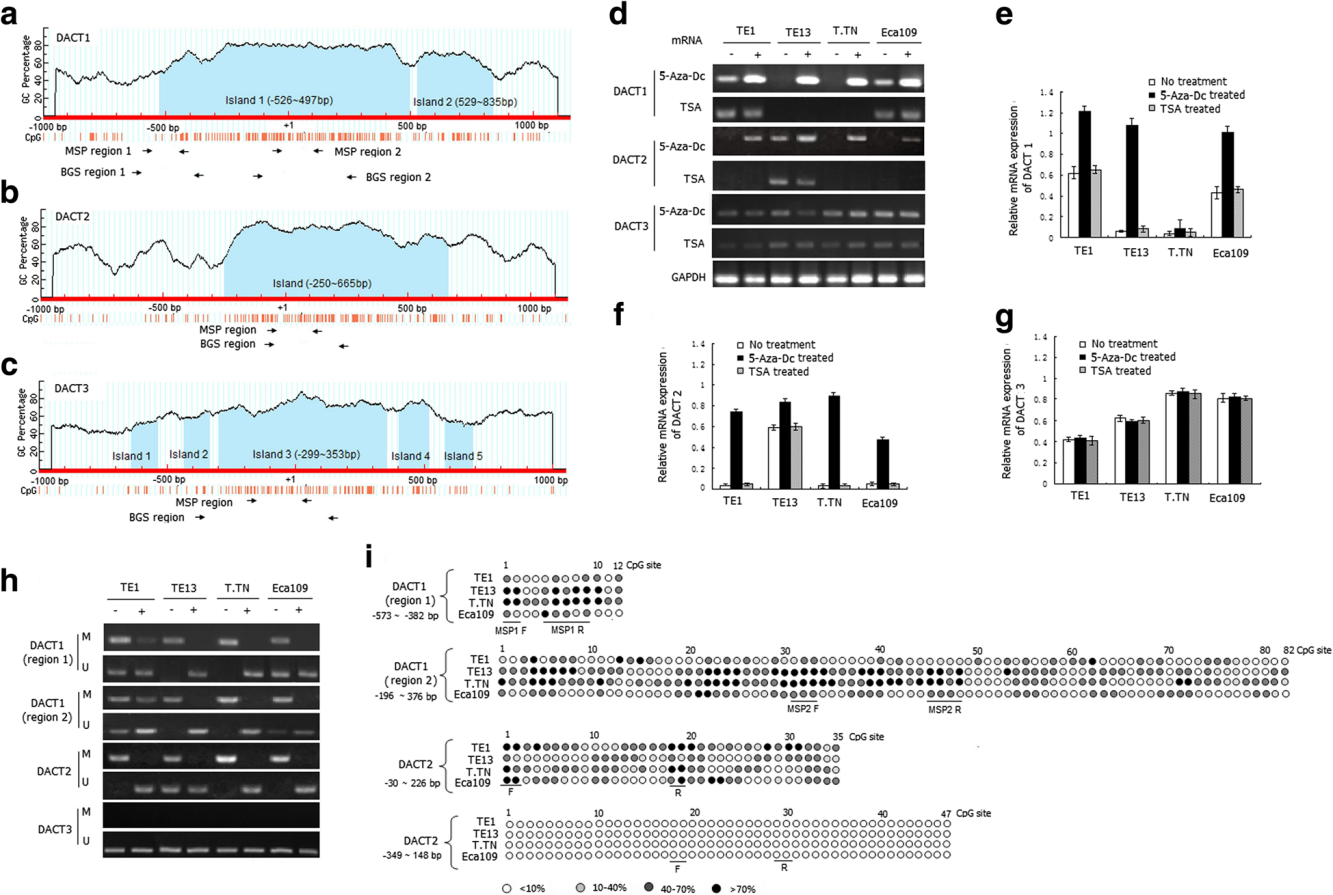
The mRNA expression and methylation analysis of *DACT1, DACT2* and *DACT3* in esophageal cancer cell lines. **a**, **b**, **c** Schematic structure of CpG islands in *DACT1, DACT2* and *DACT3* genes. The MSP and BGS-regions were shown in the schematic. The transcription start point was as +1. **d** The changes of *DACT1, 2, 3* mRNA expression in treated or untreated cells with 5-Aza-Dc or TSA by RT-PCR method; **e**, **f**, **g** Relative mRNA expression of *DACT1, 2, 3* in treated or untreated cells with 5-Aza-Dc or TSA by Quantitative realtime RT-PCR method. **h** The methylation status of *DACT1, 2, 3* in four cell lines with(+) or without(−) 5-Aza-Dc treatment detected by MSP method. M, methylated gene; U, unmethylated gene. **i** The methylation status of every CpG site in two regions of *DACT1*, one region of *DACT2* and one region of *DACT3* in four tumor cell lines by BGS method. The color of circles for each CpG site represents the percentage of methylation. The “F” and “R” represent forward and reverse primers for MSP, respectively

Total DNA was isolated from cell lines using DNAzol (Invitrogen, Carlsbad, CA, USA) and the genomic DNA from tissues of ESCC was prepared using the Proteinase K digestion method. Genomic DNA was treated with Epitect Fast Bisulfite Conversion Kits (Qiagen, Germany) according to the manufacturer’s instructions. Unmethylated cytosines was converted to uracil by bisulfite treatment, whereas methylated cytosine couldn’t be converted and remained as cytosine. Based on this potential difference in the DNA sequence between methylated and unmethylated alleles after bisulfite treatment, we designed MSP primers to analyze two regions of *DACT1*, and one region of *DACT2 and DACT3*. One of the regions in *DACT1* was encompassing TSS (region 2, from −16 ~ 105 bp) and the other was located in the CGI shore (region 1, from −540 ~ −419 bp), which was the flank of the traditional CpG islands and had relatively low CpG density. The methylation status of *DACT2* (from −30 ~ 126 bp) and *DACT3* (from −154 ~ 28 bp) were determined in the region encompassing TSS (Fig. 1a, b, c). The MSP products were analyzed on 2% agarose gels with ethidium bromide and visualized under UV illumination. Genomic DNA, which was treated by CpG methyltransferase (Sss I) following the manufacturer’s directions (New England BioLabs, Inc, Beverly, MA), was used as positive control. Water blank was used as a negative control. Every sample was repeated methylation analysis in duplicate in order to ensure the quality control. For BGS, primers were designed to recognize sodium bisulfite converted DNA and encompassing the MSP regions (*DACT1*, BGS region 1 from −573 ~ −382 bp, BGS region 2 from −196 ~ 376 bp; *DACT2*, from −30 ~ 226 bp; *DACT3*, from −349 ~ 148 bp) (Fig. 1a, b, c). The primer sequences of *DACT1, DACT2, DACT3* and PCR conditions (product size and annealing temperatures) were shown in Additional file 1: Table S1. The target fragment of PCR products for BGS were purified and cloned into pGEM-T vectors (Promega, San Luis Obispo, CA) and 10 clones of each specimen were sequenced by automated fluorescence-based DNA sequencing.

### Statistical analysis

Statistical analysis was performed using SPSS19.0 software package (SPSS Company, Chicago, IL). The methylation frequency of *DACT1, DACT2, DACT3* between ESCC and corresponding adjacent tissues were compared using Chi-square test. The mRNA expression of *DACT* genes was expressed as the mean ± standard deviation and the means were compared using the Student’s *t* test. Kaplan–Meier survival curves were constructed and the Log-rank tests were used as needed for the univariate comparison of *DACT* expression and methylation categories. Cox’s multivariate test was used to adjust for potentially confounding variables and to evaluate the role of *DACT* as an independent predictor for patient prognosis. Two-sided tests were used to determine significance and *P* values < 0.05 was regarded as statistically significant for all statistic tests.

## Results

### Frequent silencing of *DACT1, DACT2* and *DACT3* in esophageal cancer cell lines

The mRNA expression of *DACT1, DACT2* and *DACT3* were examined in four esophageal cancer cell lines by RT-RCR and qRT-RCR methods. The mRNA expression of *DACT1* was silenced in TE13, T.Tn cell lines and reduced in TE1, Eca109 cell lines; the mRNA expression of *DACT2* was silenced in TE1, T.Tn, Eca109 cell lines and reduced in TE13 cell line; while the mRNA expression levels of *DACT3* remained relatively weak in all four cell lines (Fig. 1d, e, f, g).

### Up-regulation of *DACT1* and *DACT2*, but not *DACT3*, by 5-Aza-Dc treatment in esophageal cancer cell lines

In order to clarify the possible epigenetic mechanism of down-regulation of *DACT1, DACT2* and *DACT3* expression (such as methylation and acetylation), we further detected the mRNA expression of *DACT1,2,3* in cell lines after 5-Aza-Dc (DNA methyltransferase inhibitor) or TSA (histone deacetylase inhibitor) treatment. As shown in Fig. 1d, e, f, g, the mRNA expression of *DACT1* and *DACT2* were all significantly increased in four esophageal cancer cells after treatment with 5-Aza-Dc, but not with TSA. However, *DACT3* mRNA was not significantly up-regulated after treatment with 5-Aza-Dc or TSA in four esophageal cancer cell lines.

### The aberrant promoter methylation of *DACT1* and *DACT2* induce down-regulation of their mRNA expression in esophageal cancer cell lines

To investigate whether the silencing of *DACT1, 2, 3* were associated with their hypermethylation, the MethPrimer program [27] and the CpG island searcher [28] were firstly used to analyze the CpG islands within the sequence of *DACT* genes. As shown in Fig. 1, *DACT1, DACT2* and *DACT3* were all shown to have the CpG islands. MSP results were shown in Fig. 1h, complete methylation was found in TE13 and T.TN cell lines and incomplete methylation was observed in TE1 and Eca109 cell lines in the two regions of *DACT1*. The complete methylation of *DACT2* was detected in TE1, T.TN, Eca109 cell lines and incomplete methylation was detected in TE13 cell lines. No methylated bands of *DACT3* were detected in all four cell lines. The BGS assay was used to reveal dense methylation of every CpG sites. As shown in Fig. 1i, the methylation frequency of *DACT1* CpG sites were higher in TE13 and T.TN cell lines than those in TE1 and Eca109 cell lines, especially the sites which were contained in the primers. The methylation frequency of *DACT2* CpG sites were higher in TE1, T.TN and Eca109 cell lines than those in TE13 cell lines, especially the sites which were contained in the primers. No methylation was found of *DACT3* in these four cell lines. The results indicated that the MSP assay results accurately represent *DACT1, DACT2* and *DACT3* methylation status in these cell lines. After treatment with 5-Aza-Dc, demethylation of *DACT1* and *DACT2* were observed in these cells, together with the results of *DACT1* and *DACT2* expression was restored or increased after 5-Aza-Dc treatment, indicating that *DACT1* and *DACT2* expression was regulated by the methylation status of the genes.

### Inhibition of proliferation and migration in TE1 and TE13 Cell lines after restoration of *DACT1/DACT2* or treatment with 5-Aza-Dc

**Fig. 2:**
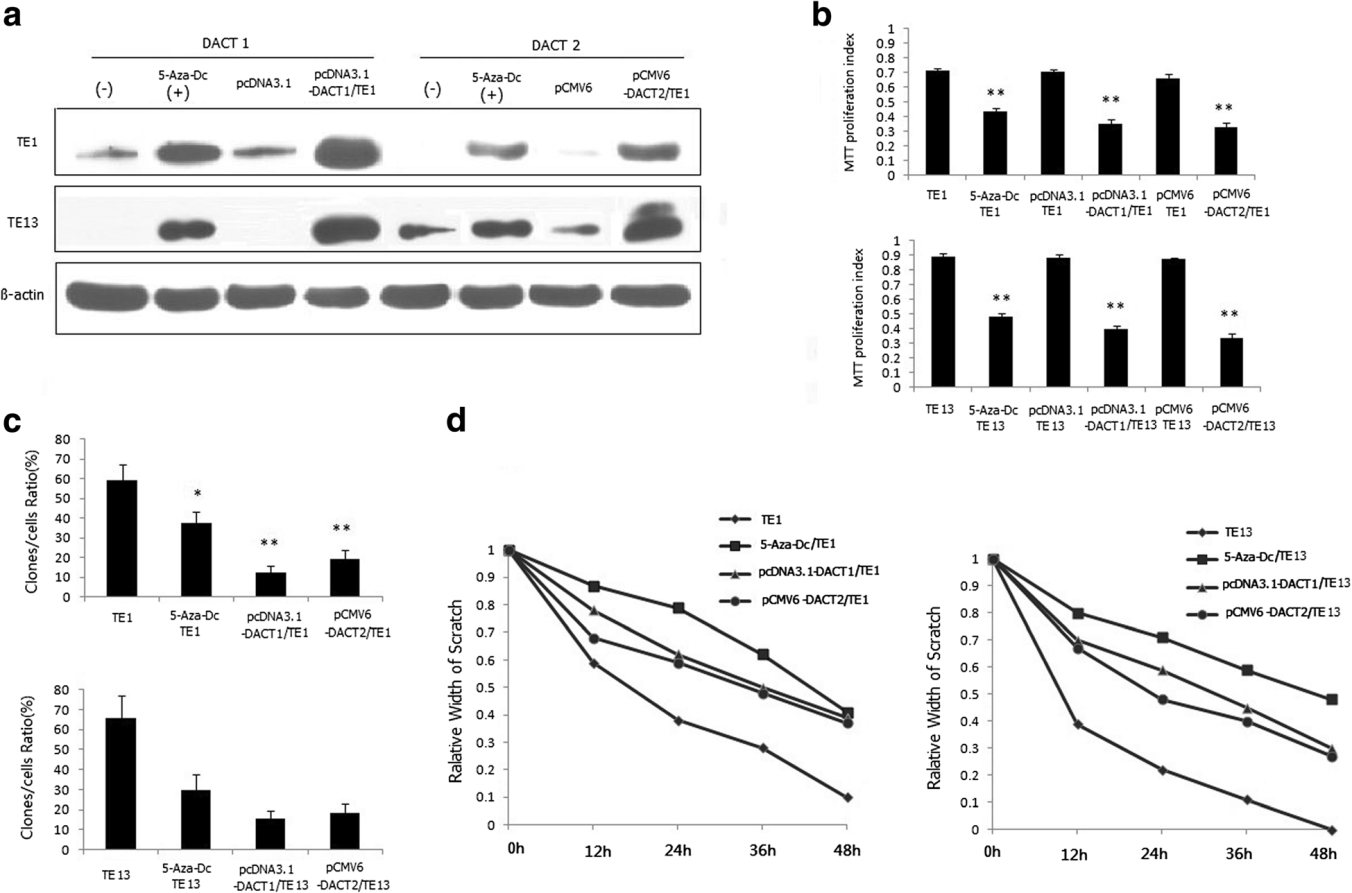
The effect on cell proliferation, colony formation and immigration abilities in TE1 and TE13 cell lines after treated with 5-Aza-dC or stable transfected of DACT1 /DACT2. **a** The protein expression of DACT1 and DACT2 in TE1 or TE13 cells after stable transfected with *DACT1*/*DACT2* plasmid or treated with 5-Aza-dC using western-blot method. **b** The results of MTT assay in treated or untreated TE1/TE13 cells with 5-Aza-dC or the stable transfected TE1/TE13 cells; **Compared with untreated cell line, *P* < 0.01; **c** The results of colony formation assays in treated or untreated TE1/TE13 cells with 5-Aza-dC or the stable transfected TE1/TE13 cells; **Compared with untreated cell line, *P* < 0.01; **d** The relative scratch width of treated or untreated TE1/TE13 cells or the stable transfected TE1/TE13 cells detected by Wound healing analysis. The initial width of the scratch was as 1

TE1 and TE13 cells, which demonstrated low or negative expression of *DACT1* and *DACT2* genes, were adopted to transfect with *DACT1* or *DACT2* plasmid and investigate effect of *DACT1* or *DACT2* on the cell proliferation using MTT and Colony-formation assays respectively. As shown in Fig. 2, stable transfection of *DACT1/DACT2* or treatment with 5-Aza-Dc resulted in significant inhibition of the proliferation index and reduction of colony numbers in TE1 and TE13 cell lines. The Wound-healing assay was further employed to evaluate the effect of *DACT1* and *DACT2* on cell migration. Cell migration ability was significantly decreased at 12, 24, 36 and 48 h after transfection with *DACT1/DACT2* or treatment with 5-Aza-Dc (Fig. 2d). These results suggested that *DACT1* and *DACT2* may act as tumor suppressor genes in esophageal cancer through inhibition of cell proliferation and migration.

### Decreased mRNA and protein expression of *DACT1, DACT2, DACT3* in ESCC

**Fig. 3:**
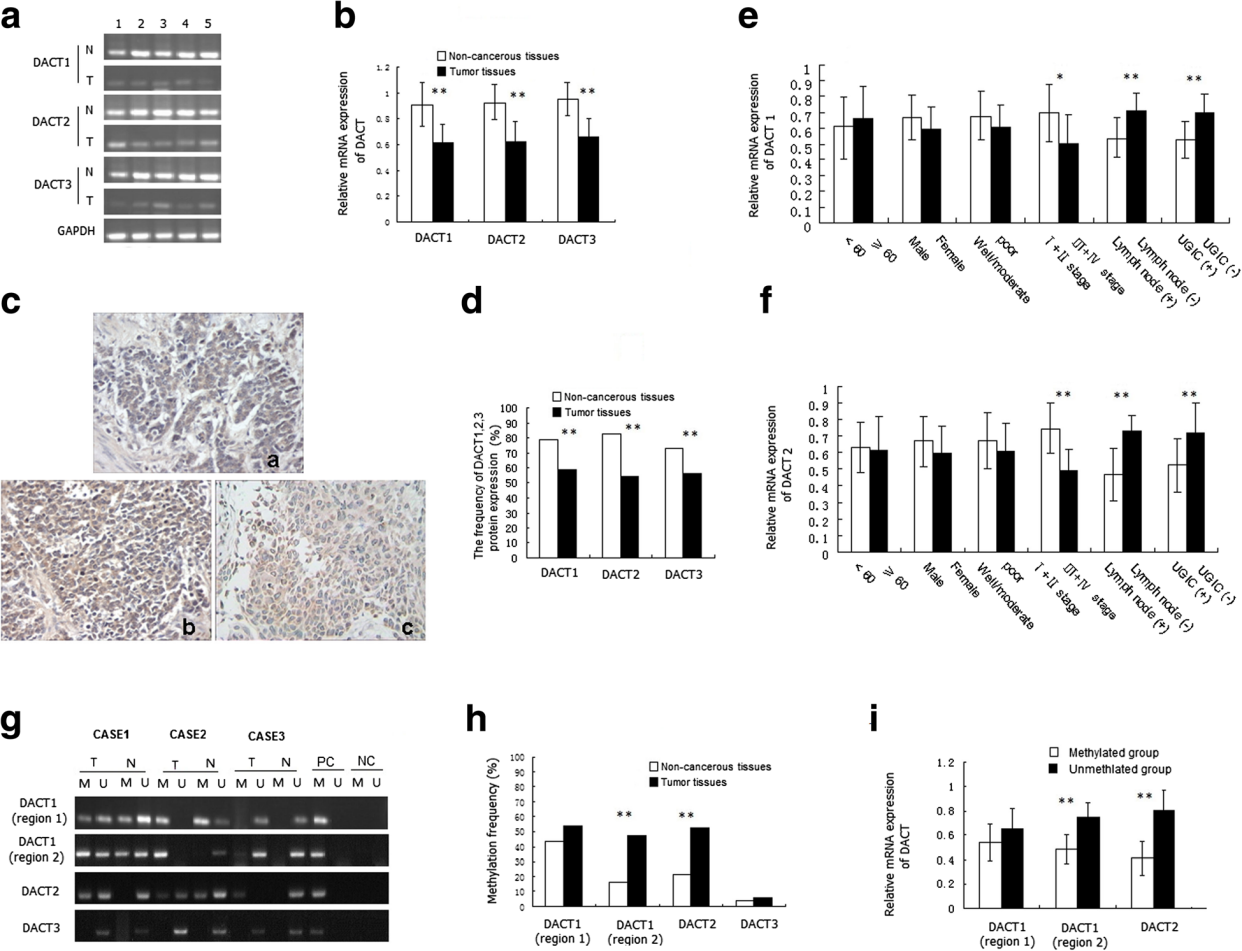
The expression and methylation analysis of *DACT1, DACT2* and *DACT3* in ESCC and corresponding adjacent non-cancerous tissues. **a** Representative RT-PCR results of *DACT1, 2, 3* in 5 matched pairs (case1-case5) of ESCC tissues (T) and non-cancerous tissues (N). GAPDH was used as an endogenous control. **b** Relative mRNA expression of *DACT1, 2, 3* in ESCC and adjacent tissues. **c** Representative immunohistochemical staining of *DACT1, 2, 3* in ESCC tumor tissues (SP × 200). a: the positive expression of *DACT1;* b: the positive expression of *DACT2;* c: the positive expression of *DACT3;* **d** Positive expression frequency of *DACT1, 2, 3* proteins in ESCC and corresponding adjacent tissues. **e**, **f** Relative mRNA expression of *DACT1* and *DACT2* in different subgroups of ESCC cases, which was expressed as the mean ± standard deviation. ^*^ *P* < 0.05, ^**^ *P* < 0.01. **g** Representative MSP results of *DACT1, DACT2* and *DACT3* in 3 matched pairs (case1-case3) of tumor tissue (T) and non-cancerous tissues (N). M, Methylation; U, Unmethylation; PC, positive control; NC, negative control. **h** The methylation rate of *DACT1, 2, 3* in ESCC tissues and non-cancerous tissues. ^**^ *P* < 0.01. **i** The relative mRNA expression of *DACT1* and *DACT2* in methylated or unmethylated groups. The mRNA expression of *DACT1 and DACT2* were all associated with methylation status of their TSS-regions in ESCC*.* ^**^ *P* < 0.01

*DACT1, DACT2, DACT3* mRNA expression in ESCC tissues were reduced significantly compared with those in corresponding adjacent non-cancerous tissues (t_DACT1_ = 16.548, *P* < 0.001; t_DACT2_ = 7.236, *P* < 0.001; t_DACT3_ = 7.124, *P* < 0.001) (Fig. 3a, b). IHC method was used to evaluate the protein expression of DACT1, DACT2, and DACT3 in ESCC tissues. The pattern of immunohistochemical staining of them was cytoplasmic (Fig. 3c). The frequency of DACT1, DACT2, DACT3 positive expression in tumor tissues was significantly lower than that in corresponding adjacent non-cancerous tissues (Fig. 3d; *P* < 0.001). When stratified for clinicopathologic characteristics, *DACT1* and *DACT2* mRNA and protein expression was associated with clinical stage, Lymph node metastasis and UGIC family history (*P* < 0.05), but not with age, gender, histological grade (Table 1; Fig. 3e, f).

### Aberrant methylation of *DACT1* and *DACT2* in ESCC clinical specimens

The methylation analysis of *DACT1, DACT2* and *DACT3* were successfully performed uding MSP method in all specimens (Fig. 3g). The methylation frequency of *DACT1* was different in two regions (Fig. 3h). Of primary ESCC and corresponding non-cancerous tissues, hypermethylation was observed in 54.1% (86/159) and 43.4% (69/159) at region1, 47.8% (76/159) and 16.4% (26/159) at region2, respectively. The methylation frequency of *DACT1* in the region1 and region 2 were all higher than those in corresponding adjacent tissues, however, only region 2 showed the statistical difference (*P*_region1_ > 0.05; *P*_region2_ < 0.001; Fig. 3h). The methylation frequency of *DACT2* was 52.2% (83/159) in tumor tissues, which was higher than that in corresponding adjacent tissues (21.4%, 34/159, *P* < 0.001; Fig. 3h). The methylation frequency of *DACT3* was very low both in ESCC and corresponding adjacent tissues (3.8 and 5.7%; *P* = 0.598) (Fig. 3h).

When stratified for clinicopathologic characteristics, the methylation status of region1 in *DACT1* was not associated with any characteristic, and the TSS-region in *DACT1* (region2) and *DACT2* were only associated with UGIC family history. But the simultaneous methylation frequency of *DACT1* (region2) and *DACT2* were associated with clinical stage, LN metastasis and UGIC family history (Table 2).

**Table 2:** Methylation status of DACT1 and DACT2 in ESCC cases

Group	N	DACT1 (region 1)		DACT1 (region 2)		DACT2		Simultaneous methylation of DACT1 and DACT2	
		M (%)	*p*	M (%)	*p*	M (%)	*p*	M (%)	*p*
Age									
<60	74	39(52.7%)	0.744	32(43.2%)	0.283	39(52.7%)	0.906	23(31.1%)	0.574
≥60	85	47(55.3%)		44(51.8%)		44(51.8%)		30(35.3%)	
Gender									
Male	93	48(51.6%)	0.457	40(43.0%)	0.151	47(50.5%)	0.618	35(37.6%)	0.550
Female	66	38(57.6%)		36(54.5%)		36(54.5%)		18(27.3%)	
Histological grade									
Well/moderate	98	49(50.0%)	0.190	41(41.8%)	0.056	46(46.9%)	0.092	28(28.6%)	0.106
Poor	61	37(60.7%)		35(57.4%)		37(60.7%)		25(41.0%)	
Clinical stage									
I + II	86	46(53.5%)	0.869	35(40.7%)	0.052	39(45.3%)	0.060	22(25.6%)	0.015
III + IV	73	40(54.8%)		41(56.2%)		44(60.3%)		32(43.8%)	
Lymph node									
+	121	70(57.9%)	0.089	63(52.1%)	0.055	67(55.4%)	0.153	48(39.7%)	0.001
-	38	16(42.1%)		13(34.2%)		16(42.1%)		4(10.5%)	
Family history of UGIC									
+	61	38(62.3%)	0.101	36(59.0%)	0.025	40(65.6%)	0.008	29(47.5%)	0.004
-	98	48(49.0%)		40(40.8%)		43(43.9%)		25(25.5%)	

*M* methylation, *U* unmethylation

### Association between expression and methylation status of *DACT1, DACT2*

As show in Fig. 3i and Table 3, the mRNA and protein expression of *DACT1, DACT2* in ESCC tissues with hypermethylation of TSS-region were significantly reduced compared to those in ESCC tissues without methylation of this region (*P* < 0.05); However, the mRNA and protein expression of *DACT1* was not different between the ESCC tissues with or without hypermethylation of CGI-shore region (region1) (*P* > 0.05). The results indicated that the reduced expression of *DACT1* and *DACT2* were associated with methylation status of the TSS-region, and the methylation status of CGI-shore region was not associated with the transcriptional inhibition of *DACT1*.

**Table 3:** Correlation between the methylation status of DACT1, DACT2 and its protein expression in ESCC cases

Gene	Positive protein expression (%)		*P*
	M group	U group	
DACT1(region 1)	53.5% (46/86)	65.8% (48/73)	0.117
DACT1(region 2)	30.3% (23/76)	85.5% (71/83)	<0.001
DACT2	34.9% (29/83)	75.0% (57/76)	<0.001

*M* methylation, *U* unmethylation

### Survival analysis of *DACT1* and *DACT2* in ESCC patients

**Fig. 4:**
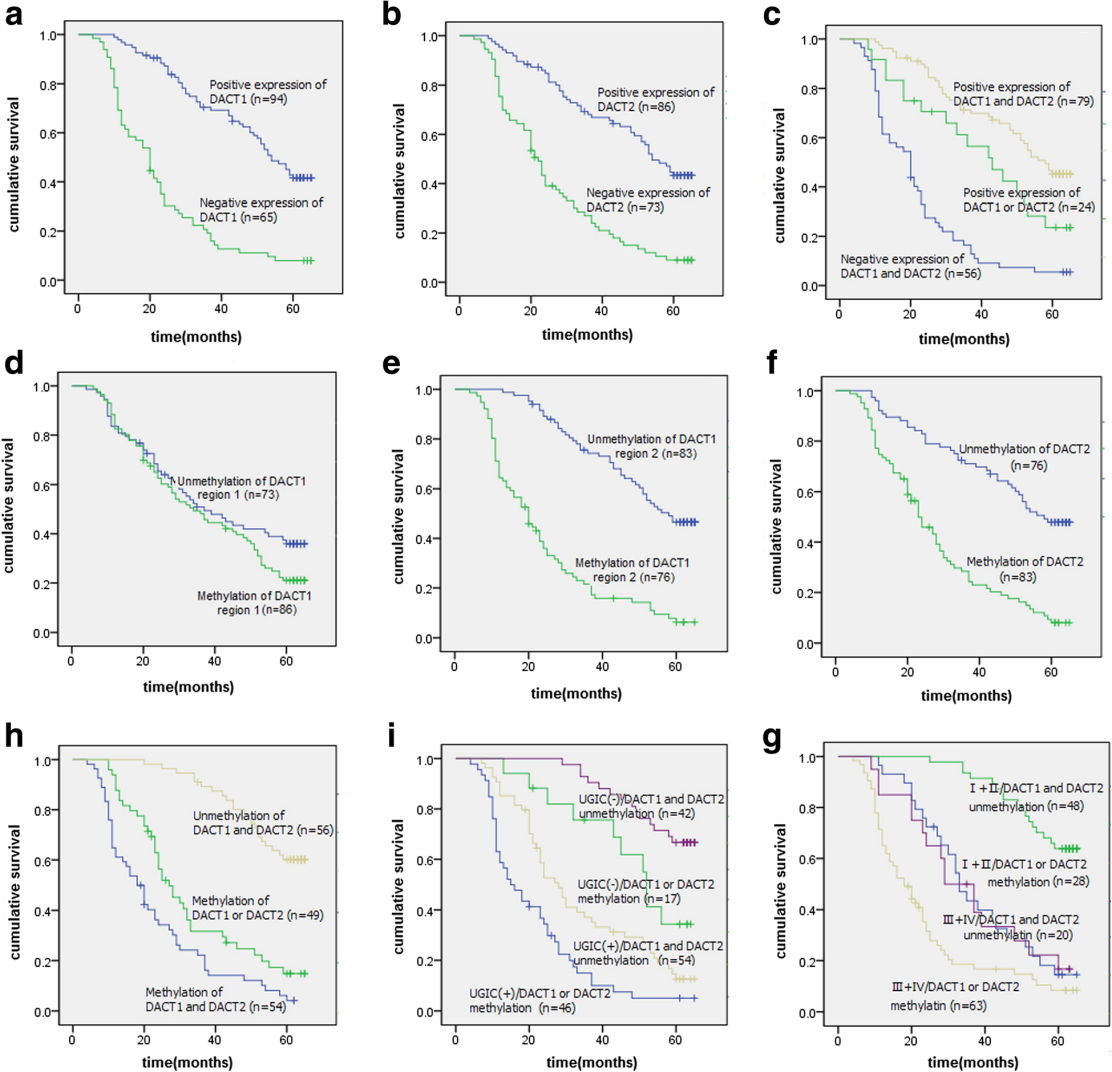
Kaplan–Meier univariate survival analysis of *DACT1 / DACT2* expression and methylation status in ESCC. **a**, **b**, **c** Kaplan–Meier curves for cumulative survival stratified by *DACT1* and *DACT2* expression: showing a direct correlation between *DACT1* or *DACT2* expression and ESCC patient’s survival. **d**, **e**, **f**, **g** Kaplan–Meier curves for cumulative survival stratified by the methylation status of two regions of *DACT1* and one region of *DACT2*: There was not correlation between the region 1 methylation status of *DACT1* and the ESCC patient’s survival. The hypermethylation of TSS-region in *DACT1* and *DACT2* were associated with the poor patient survival of ESCC cases; Simultaneous methylation of *DACT1* and *DACT2* showed worst prognosis. (H) Kaplan–Meier curves for cumulative survival stratified by methylation status and UGIC family history: ESCC patients with positive UGIC family history and *DACT1* or *DACT2* methylation showed poor patient survival. (I) Kaplan–Meier curves for cumulative survival stratified by methylation status and TNM stages: Stage III and IV ESCC patients with *DACT1* or *DACT2* methylation showed the poor patient survival

As show in Fig. 4a and b, DACT1 and DACT2 protein expression were positively correlated with ESCC patients’ survival (*P*_DACT1_ < 0.001, *P*_DACT2_ = 0.001, Log-rank test). The simultaneous positive expression of DACT1 and DACT2 showed the best prognosis (*P* < 0.001, Log-rank test, Fig. 4c). The methylation status of region1 in *DACT1* was not correlated with ESCC patients’ survival (*P* = 0.105, Log-rank test; Fig. 4d) and TSS-region methylation status of *DACT1* and *DACT2* were inversely correlated with ESCC patients’ survival (*P*_*DACT1*_ < 0.001, *P*_*DACT2*_ < 0.001, Log-rank test; Fig. 4e, f). Furthermore, the ESCC patients with simultaneous methylation of *DACT1* and *DACT2* showed worst prognosis (*P* < 0.001, Log-rank test; Fig. 4g). ESCC patients in stage III / IV or with positive UGIC family history, and with *DACT1* or *DACT2* methylation showed poor prognosis (Fig. 4h and i, *P* < 0.001, Log-rank test). To determine which variables were independent predictors of ESCC patients’ survival, a multivariate analysis was performed using COX proportional hazard regression model. The results indicated that the methylation status of TSS-region in *DACT1* or *DACT2*, the protein expression of *DACT2*, TNM stage and UGIC family history were independently associated with ESCC patients’ survival (Table 4).

**Table 4:** Multivariate analysis of survival in ESCC cases (Cox’s test)

Variable	B	SE	*P*	Odds ratio (95% CI)
MSP1 methylation of DACT1	−0.240	0.215	0.265	0.787(0.516–1.200)
MSP2 methylation of DACT1	0.744	0.333	0.026	2.104(1.095–4.043)
methylation of DACT2	1.257	0.288	0.000	3.515(1.998–6.183)
DACT1 protein	0.520	0.298	0.081	1.681(0.937–3.016)
DACT2 protein	−0.804	0.261	0.002	0.448(0.269–0.747)
TNM stage	1.533	0.222	0.000	4.632(3.000–7.152)
Family history of UGIC	0.737	0.207	0.000	2.089(1.391–3.137)

## Discussion

In the present study, we found the expression of *DACT1, DACT2* and *DACT3* were frequently silenced or decreased in esophageal cancer cell lines. Up-regulation of *DACT1* and *DACT2* but not *DACT3* after treatment with 5-Aza-Dc, inferring that DNA methylation may be the main regulatory mechanism for the inactivation of *DACT1* and *DACT2* but not *DACT3* in esophageal cancer cell lines, which further confirmed by direct BGS and MSP analysis of promoter methylation status of *DACT1, 2, 3*. However, the mRNA expression of the three genes was not significantly up-regulated after treatment with TSA in esophageal cancer cell lines, indicating that histone acetylation may not play crucial roles in the inactivation of *DACT1, DACT2* and *DACT3*. The similar results were observed in other carcinomas such as *DACT1* in breast cancer [16] and *DACT2* in hepatocellular carcinoma [15], gastric cancer [29], lung cance [17], colon cancer [13], and also in esophageal cancer [30]. But the result about *DACT3* was different from the previous studies. The histone modification may be the main regulated mechanism of *DACT3 in* colorectal cancer [18]. The difference may partly due to the fact that histone modification has the tumor cell specific and the precise mechanism of this gene inactivation need to be further studied. In addition, the inhibition of proliferation and migration was further detected in TE1 and TE13 cells after treatment with 5-Aza-Dc or stable transfection of *DACT1* or *DACT2* plasmid, indicating the tumor suppressor role of *DACT1* and *DACT2* in esophageal cancer cell lines.

Aberrant methylation of CpG islands in gene promoter has been well-established as a major mechanism for the inactivation of tumor suppressor genes in tumorigenesis [31–33]. The hypermethylation of *DACT1* in the region near TSS within CpG islands was detected in multiple breast cancer cell lines and primary breast tumors, and the methylation status in this region was a main epigenetic mechanism of *DACT1* silencing in breast cancer [16]. Meanwhile, recent studies points out that DNA methylation can directly silence genes with non-CpG islands, such as CGI shore [34, 35]. The aberrant methylation of *DACT1* in CGI shore region had been detected in primary gastric cancer and the methylated CpG site count in this region had the significant applicability for prognosis evaluation of gastric cancer patients [36]. Two regions including TSS-region and CGI shore-region were selected to analyze the methylation status of *DACT1* in present study. The TSS-region methylation frequency of *DACT1* in tumor tissues was significantly higher than that in adjacent tissues and was associated with the decreased mRNA and protein expression of *DACT1*. The methylation frequency of CGI shore in *DACT1* was higher both in tumor and adjacent tissues. These results suggested that hypermethylation of TSS-region was more cancer-specific and was associated with the transcriptional inhibition of *DACT1.* The TSS-region may be regarded as the critical CpG region of *DACT1*. The CGI shore-region was more likely to be methylated but may be not necessary for the tumor formation. The TSS-region was selected to analyze the methylation status of *DACT2* and there was a significant concordance between the hypermethylation of this gene and its reduced expression in the present study. By using the same MSP primers as we used in this study, Yu et al. [29] found *DACT2* was frequently methylated in human gastric cancer and aberrant methylation may be a main mechanism of *DACT2* inactiviton. The similar results were also reported in hepatocellular carcinoma [15], lung cancer [17], colon cancer [13] and also in esophageal cancer [30]. When stratified for clinicopathologic characteristics, the methylation status of *DACT1* or *DACT2* was only associated with UGIC family history, but the simultaneous methylation of *DACT1* and *DACT2* were associated with clinical stage, LN metastasis and UGIC family history, suggesting that multiple gene methylation may be an ideal cancer biomarker in progression of ESCC and may have definite value on estimating prognosis of ESCC patients.

The relationship between *DACT1/DACT2* protein expression, methylation status and the 5-year overall survival rate was further analyzed to evaluate their value in ESCC patients’ prognosis. In the present study, we showed that protein expression and methylation status of *DACT1* and *DACT2* were significantly and directly correlated with ESCC patients’ survival, suggesting that the inactivation of *DACT1* and *DACT2* via hypermethylation may confer a growth advantage in ESCC. Therefore, the hypermethylation and inactivation of *DACT1* and *DACT2* may be considered to be the poor prognostic factors to ESCC patients. But the methylation status of CGI shore-region in *DACT1* was not associated with ESCC patients’ survival, mainly due to the reason that this region was not the critical CpG region of *DACT1*. This result was different from a previous study [36] and the further studies with a larger number of patients may be needed to verify the results. Furthermore, the survival rate of ESCC was closely associated with the stage in which the malignancy is diagnosed [37]. The stage III/IV ESCC patients with *DACT1* or *DACT2* methylation showed the worse survival, which further indicated the methylation status of *DACT1* and *DACT2* could be considered as the useful markers for evaluating the prognosis of ESCC. UGIC family history was one of the important factors on the occurrence of ESCC in the high incidence regions of North China [6, 23, 38]. In the present study, the ESCC patients both with positive UGIC family history and *DACT1/DACT2* methylation had been showed the worst survival, indicating the poorer prognosis of *DACT1/DACT2* methylation in the high risk populations. In multivariate analysis, the methylation status of TSS-region in *DACT1* and *DACT2*, negative expression of *DACT2*, tumor stage, and positive UGIC family history provided independent predictive information on ESCC patients’ poor survival.

## Conclusion

In conclusion, the present study suggests that the TSS region hypermethylation may be one of the main mechanisms for reduced expression of *DACT1* and *DACT2* in ESCC. The CGI shore region in *DACT1* is more likely to be methylated but is not cancer-specific and isn’t related to the transcriptional inhibition of *DACT1*. The simultaneous methylation of *DACT1* and *DACT2* may play important roles in progression of ESCC, and may serve as prognostic biomarkers for ESCC patients.

## Additional file

Additional file 1: Table S1. Primer sequences, annealing temperature and product size of DACT gene Family. (DOC 56 kb)
