# Supplementary material for: Aberrant methylation of DACT1 and DACT2 are associated with tumor progression and poor prognosis in esophageal squamous cell carcinoma
Source: J Biomed Sci. 2017 Jan 11;24:6. doi: 10.1186/s12929-016-0308-6 (PMC5225534; doi:10.1186/s12929-016-0308-6)
Supplement: Additional file 1: Table S1. — Primer sequences, annealing temperature and product size of DACT gene Family. (DOC 56 kb) [file 12929_2016_308_MOESM1_ESM.doc]

**Table S1 Primer sequences, annealing temperature and product size of DACT gene Family**

| Types | Primer sequence | Annealing temperature (℃) | Product size(bp) |
| --- | --- | --- | --- |
| DACT1 |  |  |  |
| Region1-methylation | F: 5’- CGGTGTGAGTGGAAATGAGGAGTGGTC -3’ | 56 | 121 |
|  | R:5’- ACAAAAACCGCGACGAAACGCG -3’ |
| Region1-unmethylation | F: 5’- TGGTGTGAGTGGAAATGAGGAGTGGTT -3’ | 56 | 121 |
|  | R:5’- ACAAAAACCACAACAAAACACA -3’ |
| Region2-methylation | F: 5’- CGGGATAGTAGTAGTCGGC -3’ | 59 | 121 |
|  | R:5’- AAACGCTAAAACTACGACCGCG -3’ |
| Region2-unmethylation | F: 5’- TGGGATAGTAGTAGTTGGT -3’ | 58 | 121 |
|  | R:5’- AAACACTAAAACTACAACCACA -3’ |
| BGS region 1 | F: 5’- ATATTTTGTTTGGGAAGTGAAAG -3’ | 56 | 191 |
|  | R:5’- CTAAAACCCCAACATCCTATTACAA -3’ |
| BGS region 2 | F: 5’- TtgaTagaggaggggaagTTtg -3’ | 55 | 572 |
|  | R:5’- CCCCTAACCAACAACTCTTAA -3’ |
| RT-PCR | F: 5’- CACAAGCGAACTGACTACCG -3’ | 55 | 237 |
|  | R: 5’- GTAATTGCTCTGCTCGTCCT -3’ |
| DACT2 |  |  |  |
| Methylation | F: 5’- gattttagtttattttggcgattTGC -3’ | 55 | 148 |
|  | R:5’- CACATCTCCCGAACAAAATCCCG -3’ |
| Unmethylation | F: 5’- gattttagtttattttggcAattTGC -3’ | 54 | 148 |
|  | R:5’- CACATCTCCCAAACAAAATCCCA -3’ |
| BGS | F: 5’- TGGTTATAGATTTTAGTTTATTTGG -3’ | 56 | 256 |
|  | R:5’- CAACCCCTACAACTCCTACAAC -3’ | 56 |  |
| RT-PCR | F: 5’- ACTATGGACGAGGCAACA -3’ | 57 | 335 |
|  | R: 5’- GGTGGACTCAGAACAGGA -3’ |
| DACT3 |  |  |  |
| Methylation | F: 5’- AGTTTTCGTTAGGAAGTTTATTCG -3’ | 55.5 | 126 |
|  | R:5’- TATCACCGTCTCATATACATAAACG -3’ |
| Unmethylation | F: 5’- AGTTTTTGTTAGGAAGTTTATTTG -3’ | 54.5 | 125 |
|  | R:5’- ATCACCATCTCATCTACATAAACACC -3’ |
| BGS | F: 5’- agagggtggaaTTtgTtgTagg -3’ | 54.5 | 497 |
|  | R:5’- ACCAAACTACCCTCCAACCAA -3’ |  |  |
| RT-PCR | F: 5’- AGGATTCGGAGGTAGAAGA -3’ | 57 | 161 |
|  | R: 5’- GGAGTCACCATCGTCATAA -3’ |
| GAPDH1 | F: 5’-AGGTGAAGGTCGGAGTCAACG-3’ | 56.7 | 104 |
|  | R: 5’-AGGGGTCATTGATGGCAACA-3’ |
